# An entosis-informed prognostic index for survival stratification and molecular characterization in lung adenocarcinoma

**DOI:** 10.3389/fcell.2026.1943127

**Published:** 2026-10-05

**Authors:** Yinqin Liu, Wen Zhao, Shuyi Mu, Silun Liang, Yanping Wen, Shaobin Li, Chengyan Zhan, Yanqi Cheng, Ling Tang, Yuqin Wu, Ziyou Lin, Hong Fang, Yubin Ding

**Affiliations:** 1 Department of Traditional Chinese Medicine Preventive Healthcare, Longhua Hospital Shanghai University of Traditional Chinese Medicine, Shanghai, China; 2 Department of Thoracic Surgery, Tongren Hospital Shanghai Jiao Tong University School of Medicine, Shanghai, China; 3 Institute of Respiratory Diseases, Longhua Hospital Shanghai University of Traditional Chinese Medicine, Shanghai, China; 4 The Fourth Clinical Medical College, Guangzhou University of Chinese Medicine, Shenzhen, China; 5 Department of Internal Medicine of Oncology and Hematology, Shenzhen Traditional Chinese Medicine Hospital, Shenzhen, China; 6 Department of Respiratory Endoscopy, Department of Respiratory and Critical Care Medicine, Shanghai Chest Hospital, Shanghai Jiao Tong University School of Medicine, Shanghai, China

**Keywords:** entosis, entotic cell death, immunoinflammatory microenvironment, lung adenocarcinoma (LUAD), predicted drug sensitivity, prognostic biomarker, RAC1

## Abstract

**Background:**

Entosis is an actomyosin-dependent cell-in-cell process that may culminate in lysosome-mediated entotic cell death and is implicated in tumor evolution. Its clinical relevance in lung adenocarcinoma (LUAD) remains unclear.

**Methods:**

To construct an Entosis-related Prognostic Prediction Index (EPPI), this study integrated TCGA-LUAD transcriptomic, clinical, and somatic mutation data. Three independent GEO cohorts provided external validation. The initial candidate gene set covered components driving cell-in-cell formation and the intracellular degradation of internalized cells. For subgroup comparison, the analytical approach incorporated survival analysis, functional enrichment, tumor microenvironment profiling, mutation analysis, immunotherapy assessment, and drug-sensitivity prediction. Tissue and cellular distributions of the EPPI genes were mapped using spatial transcriptomics and single-cell RNA sequencing. Molecular docking assessed predicted drug-target interactions. Finally, *in vitro* RAC1 knockdown experiments evaluated the cellular function of a representative EPPI gene.

**Results:**

Comprising RND3, RAC1, KRAS, CTNNA1, AURKA, and ACTB, the final EPPI consistently stratified overall survival across all cohorts and showed moderate prognostic discrimination. The high-EPPI group had greater tumor purity and mutational burden, with enhanced cell-cycle, DNA-repair, epithelial–mesenchymal transition, and NF-κB programs, whereas the low-EPPI group showed greater immune and stromal infiltration. TIDE and immunophenoscores also differed between subgroups. Drug-response models predicted greater sensitivity to cisplatin and paclitaxel in the high-EPPI group and to several targeted agents in the low-EPPI group. Molecular docking predicted RAC1–EHT-1864 and AURKA–alisertib interactions. Spatial and single-cell analyses showed heterogeneous EPPI-gene expression, with ACTB and RAC1 broadly distributed and AURKA restricted to selected populations. RAC1 depletion suppressed LUAD-cell proliferation, DNA synthesis, migration, and invasion.

**Conclusion:**

The EPPI is an entosis-informed prognostic index for survival stratification in LUAD. Based on this index, patients can be separated into subgroups with distinct proliferative features, genomic alterations, tissue compositions, and predicted drug sensitivities. These findings associate entosis-related molecular features with distinct cellular and clinical characteristics of LUAD.

## Introduction

Lung cancer consistently represents a major global public health challenge. According to recent epidemiological data (GLOBOCAN 2022), pulmonary malignancies account for the highest number of cancer-related fatalities worldwide, generating approximately 1.82 million deaths alongside 2.48 million novel diagnoses (Bray et al., 2024). Among these cases, lung adenocarcinoma (LUAD) represents the predominant histological subtype. Specifically, epidemiological records from 2022 demonstrate that this subtype encompasses nearly 60% of female and over 45% of male lung cancer incidences, underscoring its critical role in oncology research (Luo et al., 2025). Although advances in targeted interventions, immune checkpoint inhibitors, and emerging RNA-targeted therapeutic strategies have shown potential to improve survival outcomes for certain LUAD patients (Wang et al., 2024; Yu et al., 2024; Zhang et al., 2026), patient outcomes remain heterogeneous, and differences in therapeutic response and long-term survival are still evident (Kolb et al., 2024; Faehling et al., 2025). Recent studies have further highlighted that complex interactions within the tumor microenvironment, including immune regulation mediated by host-associated microbial factors, may contribute to variability in therapeutic responses and disease progression (Lin et al., 2026). TNM staging and conventional pathological indicators continue to guide clinical evaluation and treatment decision-making in LUAD (Detterbeck et al., 2024; Abolfathi et al., 2025). However, these conventional approaches cannot fully explain the differences in treatment response and survival among patients with the same clinical stage. Therefore, molecular biomarkers reflecting biologically relevant processes, including regulated cell death, cellular competition, and tumor–microenvironment interactions, may provide complementary information for LUAD risk stratification and individualized treatment planning.

Tumor development involves non-apoptotic forms of cell death and cell-in-cell processes in addition to classical apoptosis. These processes can influence tissue remodeling, clonal selection, and treatment response. Entosis is a cell-in-cell process in which a viable cell becomes internalized within another cell. Typically triggered by the loss of matrix attachment, entosis depends on direct physical contact between neighboring cells and involves cell-cell adhesion, Rho GTPase signaling, actomyosin remodeling, and mechanical competition (Overholtzer et al., 2007; Gaptulbarova et al., 2024). After internalization, the engulfed cell may remain viable, proliferate, escape the host, or undergo lysosome-mediated entotic cell death (Kim et al., 2024; Galluzzi et al., 2018). When entotic cell death occurs, the internalized cell is degraded within the vacuolar machinery of the host cell. Therefore, entosis refers to cell-in-cell internalization, while entotic cell death denotes the subsequent lysosome-dependent degradation of the internalized cell. This lysosome-dependent degradation differs from classical cell-autonomous apoptosis.

These molecular processes contribute to cell-in-cell formation and the regulation of interactions between internalized and host cells. Thus, alterations in cytoskeletal regulation, cell-cycle control, and lysosomal pathways may reflect molecular features associated with entosis-related processes and tumor progression. Importantly, the presence of homotypic internalization has been documented within non-small cell lung cancer (NSCLC) specimens, with a higher prevalence linked to reduced overall and disease-free survival (Liu X. et al., 2024). Meanwhile, entosis has been reported to induce chromosomal instability, which is linked to invasive tumor phenotypes and unfavorable prognosis in certain malignancies (Mackay et al., 2018). Entotic engulfment may interfere with host-cell division and thereby connect cell-in-cell processes with genomic instability and tumor evolution.

Although these cell-in-cell dynamics and intracellular degradation processes have been well characterized at the cellular level, their clinical significance in LUAD remains largely unknown. Profiling entosis-associated genes provides a practical means of characterizing transcriptional alterations related to these processes. Given the substantial molecular and microenvironmental heterogeneity of LUAD, a multi-gene prognostic index may facilitate survival stratification and the assessment of genomic, immune, stromal, and predicted drug-sensitivity differences across patient subgroups. Accordingly, this study developed and validated an entosis-informed prognostic index for survival stratification and molecular characterization in LUAD.

## Methods

### Dataset collection and preprocessing

The TCGA-LUAD cohort was used as the training cohort for EPPI construction. Corresponding RNA sequencing profiles, clinical annotations, and somatic mutation records were downloaded from the TCGA database (https://portal.gdc.cancer.gov/). For external verification purposes, three distinct microarray datasets, namely GSE13213, GSE26939, and GSE29016, were acquired from the GEO database (https://www.ncbi.nlm.nih.gov/geo/). Patients with available transcriptomic profiles and overall survival information were included in subsequent analyses. Individuals lacking definitive survival status or follow-up duration were excluded from subsequent evaluations. Expression datasets were annotated according to their corresponding platform information and processed using the original normalization procedures provided for each dataset. Somatic mutation data were utilized to profile mutational landscapes and calculate tumor mutational burden (TMB). An initial set of 56 entosis-related genes was curated from published studies and publicly available resources. These genes were selected based on their reported involvement in biological processes associated with entosis, including cell-in-cell formation, cell adhesion, Rho GTPase signaling, actomyosin remodeling, and lysosome-related intracellular processing (Supplementary Table S1). Given that all analyzed information originated from publicly accessible, de-identified platforms, additional institutional ethical board approval was not mandated.

### Development of the prognostic model

Initially, the compiled gene list was mapped against the TCGA-LUAD expression matrix, retaining only those with quantifiable transcript levels. Differential expression analysis was then performed among the entosis-related genes between LUAD tissues and adjacent non-tumor tissues using the limma package in R software. Statistical significance for DEGs was defined by an adjusted P-value threshold of <0.05 combined with a |log2FC| ≥ 1. Univariate Cox proportional hazards analysis was performed on the identified entosis-related DEGs to screen genes associated with overall survival. To reduce model complexity and avoid redundant predictors, the least absolute shrinkage and selection operator (LASSO) algorithm, implemented using the glmnet package in R, was subsequently applied (Simon et al., 2011). A 10-fold cross-validation approach was used to determine the optimal penalty parameter (λ). Genes retained by LASSO regression were further entered into a multivariate Cox regression model. The genes retained in the final multivariate model and their corresponding regression coefficients were used to construct the Entosis-related Prognostic Prediction Index (EPPI). Accordingly, EPPI represents a prognostic risk score derived from the expression of selected entosis-related genes and their corresponding Cox regression coefficients. For each patient, the EPPI value was calculated as:

$$EPPI_{j}=\exp\left[\sum_{i=1}^{n}\beta_{i}\left(\mathrm{Exp}r_{ij}-\bar{\mathrm{Exp}r_{i}}\right)\right]$$

Within this framework, *βᵢ* denotes the multivariate Cox regression coefficient for gene *i*, *Expr*_*ij*_ represents the normalized expression value of gene *i* in patient *j,* and ${\bar{Expr}}_{i}$ represents the reference expression value derived from the TCGA-LUAD training cohort and used for centering in the fitted Cox model (Gao et al., 2024).

### Prognostic model validation and clinical relevance assessment

The established prognostic algorithm and original regression coefficients were applied to the independent validation cohorts. In the TCGA-LUAD training cohort, patients were divided into high- and low-EPPI groups using the median EPPI score as the cutoff. In each external validation cohort, EPPI scores were calculated using the regression coefficients derived from the TCGA-LUAD cohort, and patients were stratified according to the median EPPI score within that cohort. Survival outcomes across these risk groups were compared through Kaplan-Meier analysis and log-rank testing. Time-dependent receiver operating characteristic (ROC) curves for 1-, 3-, and 5-year overall survival were generated using the timeROC package to evaluate the prognostic discrimination of EPPI. The associations between EPPI scores, survival status, and expression patterns of the six model genes were visualized using heatmaps and scatter plots. Principal component analysis was performed based on the six model genes to examine the distribution of patients in the reduced-dimensional space. These prognostic analyses were repeated in the external validation cohorts.

To further evaluate the prognostic value of EPPI in relation to conventional clinicopathological factors, multivariable Cox regression analysis was performed by incorporating the EPPI risk group together with age, sex, race, clinical stage, and TNM classification. The distribution of prognostic scores was examined across distinct tumor progression and metastasis classifications. A nomogram integrating EPPI with clinical variables was developed using the rms and regplot packages to estimate 1-, 3-, and 5-year overall survival probabilities. Calibration plots were used to evaluate the agreement between predicted and observed survival probabilities. Correlation analyses were performed to examine the associations among survival status, EPPI score, and expression levels of the six model genes.

### Functional enrichment and pathway activity analysis

Functional characterization of genes exhibiting differential expression between the high- and low-EPPI groups was performed using the clusterProfiler package in R. Gene Ontology (GO) enrichment analysis was performed for biological process, cellular component, and molecular function categories (Harris et al., 2004), and Kyoto Encyclopedia of Genes and Genomes (KEGG) enrichment analysis was used to identify related pathways (Kanehisa and Goto, 2000). Gene set enrichment analysis (GSEA) was further performed using the ranked gene list to identify pathways differentially enriched between the two EPPI groups (Subramanian et al., 2005). Furthermore, gene set variation analysis (GSVA) was used to calculate pathway activity scores for individual samples, which were subsequently compared between the high- and low-EPPI groups.

### Analysis of the immunoinflammatory microenvironment, genomic alterations, and predicted therapeutic response

The tumor immune microenvironment was evaluated across EPPI subgroups using multiple estimation algorithms. The applied tools included Estimation of Stromal and Immune cells in Malignant Tumor tissues using Expression data (ESTIMATE) (Yoshihara et al., 2013), Cell-type Identification by Estimating Relative Subpopulations of RNA Transcripts (CIBERSORT) (Chen et al., 2018), Tumor Immune Estimation Resource (TIMER) (Li et al., 2017), Estimation of Proportion of Immune and Cancer cells (EPIC) (Racle et al., 2017), and xCell (Aran et al., 2017). These algorithms were used to characterize immune-cell infiltration from complementary perspectives. CIBERSORT estimated the relative proportions of immune-cell populations, whereas ESTIMATE was used to calculate ImmuneScore, StromalScore, ESTIMATEScore, and tumor purity (Yoshihara et al., 2013). TIMER, EPIC, and xCell provided additional estimates of immune-cell abundance across EPPI subgroups. Spearman correlation analyses were performed to examine the relationships between EPPI, the six model genes, immune-cell infiltration, and immune regulatory molecules.

Somatic mutation data from the TCGA-LUAD cohort were analyzed using the maftools package in R. Mutation profiles, including variant classifications and frequently mutated genes, were compared between the high- and low-EPPI groups. TMB was defined as the total number of nonsynonymous somatic mutations per megabase of coding sequence (Fusco et al., 2021). TMB values were compared between the two EPPI groups. Patients were further stratified according to both EPPI and TMB levels, and overall survival differences among the resulting groups were evaluated using Kaplan-Meier analysis.

Immunotherapy-related characteristics were assessed using Tumor Immune Dysfunction and Exclusion (TIDE) and Immunophenoscore (IPS), which evaluate different aspects of antitumor immunity (Jiang et al., 2018; Charoentong et al., 2017). TIDE was used to assess predicted T-cell dysfunction and exclusion, whereas IPS was used to characterize tumor immunogenicity under different immune checkpoint blockade settings. IPS data were obtained from The Cancer Immunome Atlas. In silico drug sensitivity was estimated using the GDSC2 database and the oncoPredict package. Predicted IC50 values for selected chemotherapeutic and targeted agents were calculated for individual samples and compared between the high- and low-EPPI groups (Maeser et al., 2021).

### Molecular docking and spatial transcriptomic analysis

Interactions involving candidate small molecule compounds and target proteins from the EPPI model were investigated through molecular docking simulations. RAC1 was docked with EHT-1864, which was identified in the drug-sensitivity analysis, whereas AURKA was docked with alisertib, an established inhibitor of AURKA. Molecular docking was then used to examine the possible interactions within these two drug–target pairs. Ligand structures sourced from PubChem were converted into three-dimensional conformations utilizing ChemOffice 2014 and saved as mol2 files. Three-dimensional protein structures were retrieved from the RCSB Protein Data Bank (Burley et al., 2023). Prior to simulation, protein structures were prepared in PyMOL version 3.1.6 by eliminating water molecules and irrelevant heteroatoms including metal ions and co crystallized ligands. Receptor and ligand files were processed utilizing AutoDock Tools version 1.5.6 to define rotatable bonds, assign charges, and add hydrogen atoms (Morris et al., 2009). Molecular docking simulations were performed utilizing AutoDock Vina (Zhang et al., 2026). The conformation displaying the lowest predicted binding energy was selected for subsequent analysis (Eberhardt et al., 2021). The resulting binding conformations were visualized within PyMOL. Two dimensional diagrams detailing protein ligand interactions were generated utilizing LigPlot + version 2.3.1 (Laskowski and Swindells, 2011).

The spatial transcriptome dataset GSE273378 was acquired from the GEO repository. Tissue sections GSM8427429 and GSM8427438 were selected for this analysis. Spatial transcriptomic data were processed utilizing Seurat and normalized via the SCTransform method (Stuart et al., 2019; Hafemeister and Satija, 2019). Spatial transcriptomic approaches have been increasingly applied to characterize spatial heterogeneity and infer cell-type-specific molecular interactions within tissues (Hong et al., 2024; Ge et al., 2025). Computational approaches integrating histological information with spatial transcriptomic profiles have been developed to improve spatial molecular characterization and tissue microenvironment analysis (Wen et al., 2025). Spatial expression patterns of representative genes retained within the EPPI model were displayed utilizing the SpatialFeaturePlot function alongside tissue spatial coordinates and the corresponding expression matrix.

### Single-cell transcriptomic characterization of EPPI model genes

Single-cell RNA sequencing data were obtained from GEO (GSE131907) to examine the cellular expression patterns of the EPPI model genes in LUAD (Kim et al., 2020). Six primary lung tumor samples annotated as ‘tLung’ were included: LUNG_T18, LUNG_T19, LUNG_T20, LUNG_T28, LUNG_T30, and LUNG_T34. After quality control, 24,166 cells and 29,634 genes were retained.

Single-cell data were processed using Seurat (Hao et al., 2024; Zhang et al., 2024; Li et al., 2025). Expression values were normalized using LogNormalize with a scale factor of 10,000, and the top 3,000 variable genes were selected for subsequent analysis. Batch effects among samples were corrected using Harmony (Korsunsky et al., 2019). The first 30 Harmony dimensions were used for clustering and UMAP visualization, with the clustering resolution set to 0.6. Cell populations were identified based on canonical marker genes together with the original cell annotations, and cell-cycle phases were assigned using CellCycleScoring.

The expression of the six EPPI model genes was then examined across the annotated cell populations. To further characterize RAC1-associated cellular features, cells were divided into RAC1-high and RAC1-low groups according to the median RAC1 expression level. Differentially expressed genes among cell populations and between the two RAC1 groups were identified, followed by GO and KEGG enrichment analyses using clusterProfiler (Wu et al., 2021). Metabolism-related pathway activity was further assessed using AUCell and compared across cell-cycle phases and RAC1 expression groups (Aibar et al., 2017).

### Functional evaluation of RAC1 in LUAD cells

RAC1 was selected from the six EPPI model genes for functional evaluation due to its established role in Rho GTPase-mediated cytoskeletal regulation and its widespread expression in our single-cell analysis. We performed *in vitro* experiments using the human LUAD cell lines A549 and HCC827 (Hu et al., 2024). A549 cells were cultured in F12K medium, and HCC827 cells were maintained in RPMI 1640 medium. Both media were supplemented with 10% fetal bovine serum and 1% penicillin-streptomycin. Cells were continuously grown at 37 °C in a humidified atmosphere containing 5% CO_2_. To silence RAC1 expression, we utilized three independent small interfering RNAs along with a negative control sequence synthesized by GenePharma Biotechnology. Transfections were carried out using Lipofectamine 3000 at 60%–70% cell confluence with a final siRNA concentration of 50 nM. Cells were subsequently harvested 48 h post-transfection.

We verified RAC1 knockdown efficiency through quantitative real-time PCR. Total RNA was extracted using TRIzol reagent and reverse transcribed with the PrimeScript RT Reagent Kit. Quantitative amplification was performed using SYBR Green Master Mix on an ABI 7500 Real-Time PCR System. Relative RAC1 expression was determined using the comparative cycle threshold method with GAPDH serving as the internal reference (Schmittgen and Livak, 2008). Based on the qRT-PCR assessment of knockdown efficiency, si-RAC1-1 and si-RAC1-3 were selected for subsequent functional assays. All primer sequences are provided in the Supplementary Table S2.

Post-transfection cell proliferation and DNA synthesis were evaluated through CCK-8, colony formation, and EdU incorporation assays. To conduct the CCK-8 assay, 2000 cells were seeded per well in 96-well plates, and absorbance at 450 nm was measured at 24-h intervals over a 96-h period. Colony formation capacity was assessed by culturing 500 cells per well in six-well plates for 10–14 days. Colonies containing more than 50 cells were then fixed, stained with crystal violet, and counted. DNA synthesis was determined by incubating cells with 50 μM EdU for 2 h. This step was followed by fixation, permeabilization, EdU staining, and DAPI counterstaining.

Transwell assays were employed to investigate cell migration and invasion. To assess migration, 5 × 10^4^ transfected cells suspended in serum-free medium were seeded into uncoated upper chambers. Medium containing 10% fetal bovine serum was added to the lower chambers to serve as a chemoattractant. Migrated cells on the lower surface were fixed, stained, and quantified after 24 h. Invasion assays followed a similar protocol, where 1 × 10^5^ transfected cells were seeded into Matrigel-coated upper chambers. Invaded cells were subsequently quantified after 24–48 h.

### Statistical analysis

All statistical analyses were conducted in R version 4.4.1. We applied the Wilcoxon rank-sum test to compare two independent groups and the Kruskal–Wallis test for comparisons involving three or more groups. Welch’s t-test was used to compare the expression differences of the six EPPI model genes between the high- and low-EPPI groups. Spearman’s rank correlation determined the associations between continuous variables.

*In vitro* experimental results represent the mean ± standard deviation (SD) of three independent replicates. One-way analysis of variance (ANOVA) assessed overall group differences for the qRT-PCR, colony formation, EdU incorporation, migration, and invasion assays. Dunnett’s multiple-comparison test subsequently compared each treatment condition to the si-NC reference within individual cell lines. CCK-8 time-course data were analyzed using two-way ANOVA with Dunnett’s test to compare each RAC1-silenced group with the control at each time point. All statistical evaluations were two-sided. A *P* value below 0.05 defined statistical significance.

## Results

### Development of an entosis-informed prognostic index in LUAD

Figure 1 illustrates the comprehensive analytical workflow of this study. Initial screening within the TCGA-LUAD cohort identified eleven entosis-related genes significantly associated with overall survival (Figure 2A). LASSO Cox regression further reduced the candidate set (Figures 2B,C). Multivariable Cox analysis identified six genes, RND3, RAC1, KRAS, CTNNA1, AURKA, and ACTB, for EPPI construction (Figure 2D; Supplementary Table S3).

**FIGURE 1:**
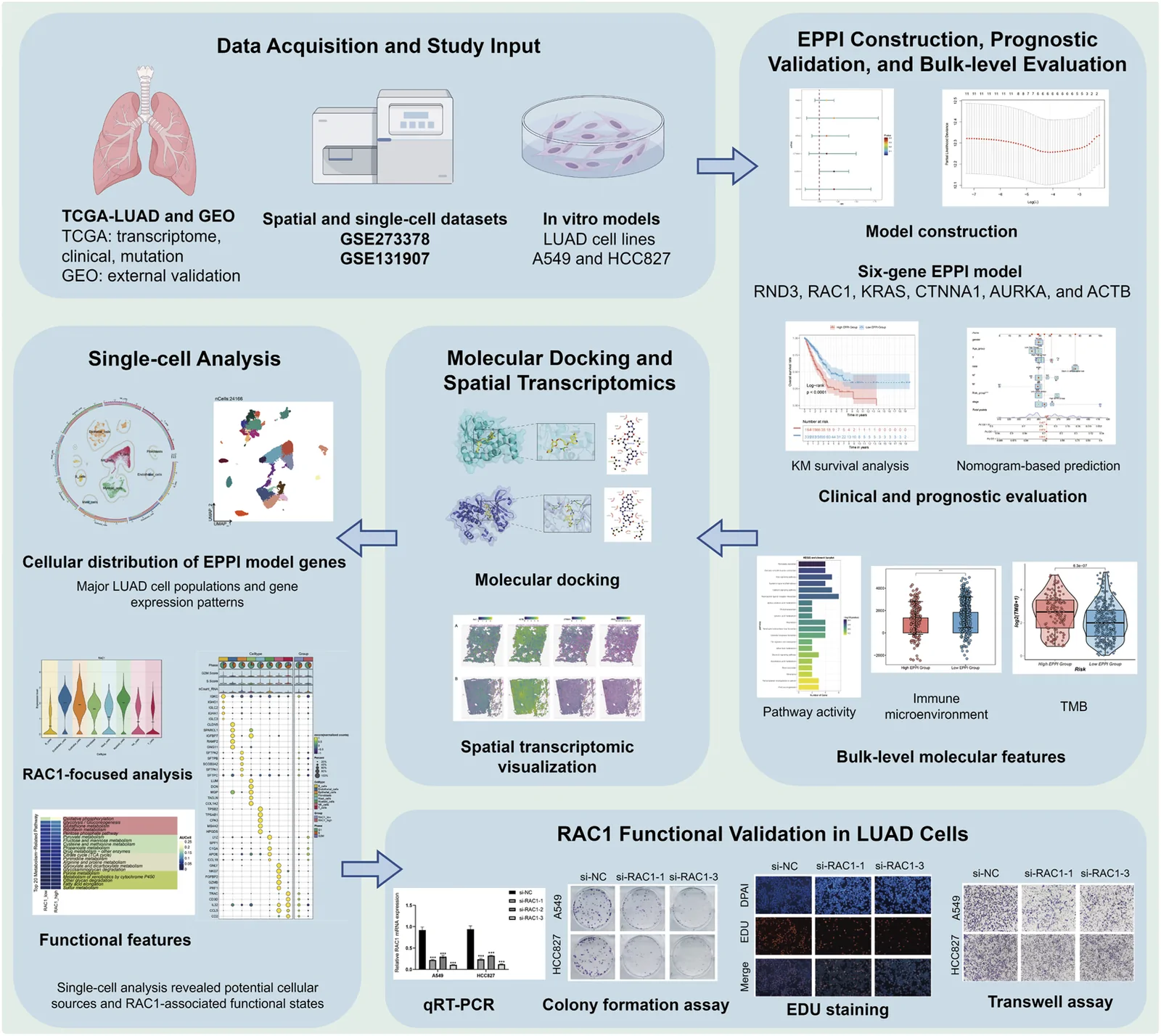
Overview of EPPI development and subsequent analyses. TCGA-LUAD data were used for EPPI construction, and three GEO cohorts were used for external validation. Molecular and functional characterization included genomic, immune, drug-sensitivity, spatial transcriptomic, single-cell, molecular docking, and *in vitro* analyses.

**FIGURE 2:**
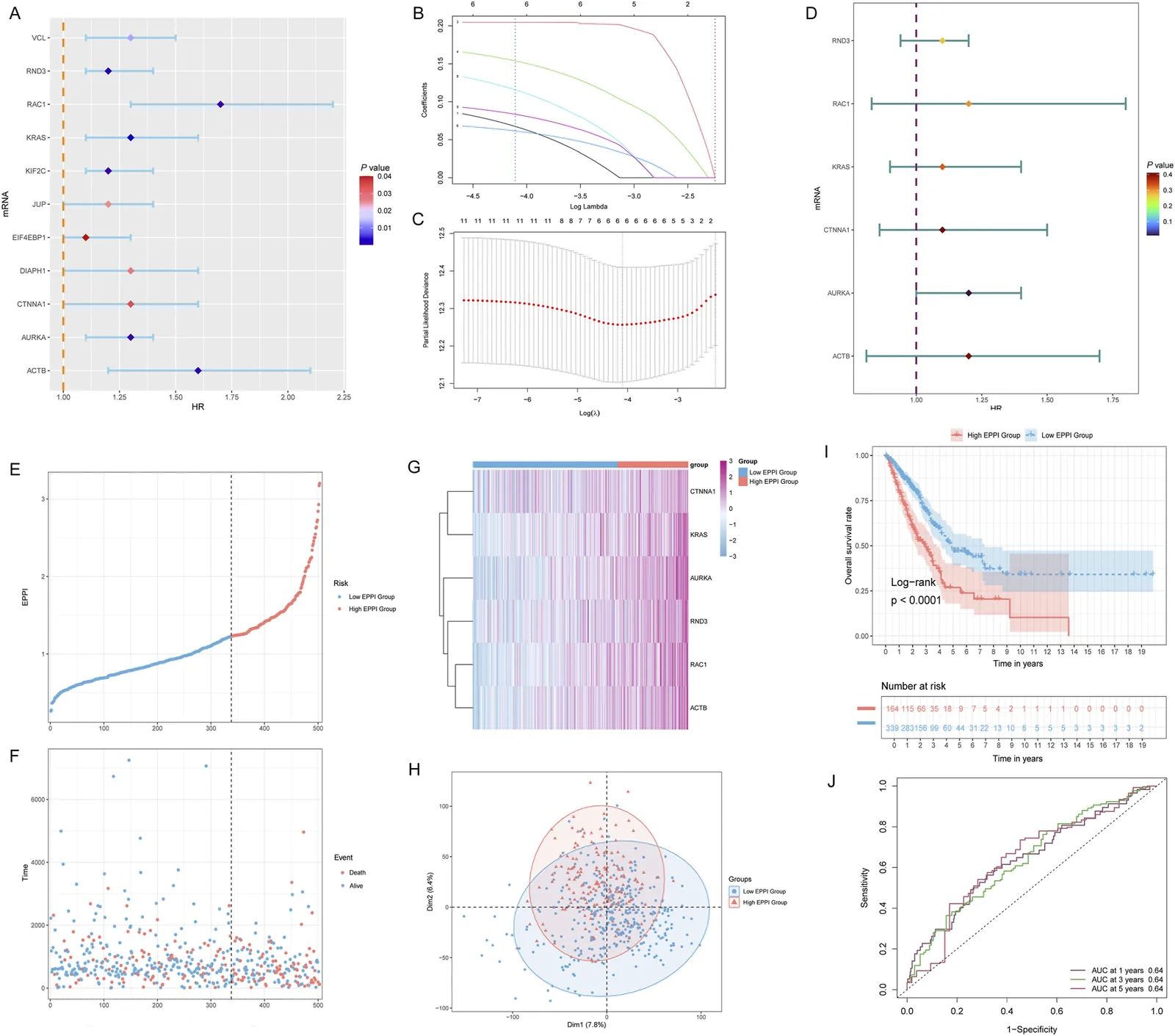
Establishment and validation of EPPI in the TCGA cohort. **(A)** Prognostic relevance of entosis-related genes evaluated using Cox regression. **(B,C)** LASSO coefficient trajectories and cross-validation error curve. **(D)** Multivariable Cox regression coefficients for the six model genes. **(E,F)** Distribution of EPPI scores and survival status in the TCGA-LUAD cohort. **(G)** Expression patterns of the six model genes between the high- and low-EPPI groups. **(H)** Principal component analysis based on the expression of the six model genes. **(I)** Kaplan–Meier survival curves comparing the high- and low-EPPI groups. **(J)** Time-dependent ROC curves for 1-, 3-, and 5-year overall survival.

### Risk stratification and validation of the EPPI

Applying the median EPPI threshold stratified the primary TCGA-LUAD cohort into distinct high- and low-EPPI groups (Figure 2E). Survival outcome comparisons revealed a higher proportion of deaths among patients with elevated EPPI values (Figure 2F). Furthermore, the six model genes comprising RND3, RAC1, KRAS, CTNNA1, AURKA, and ACTB exhibited distinctly different expression patterns between these two risk categories (Figure 2G). Supplementary Figure 1 details these specific transcriptomic variations. Principal component analysis showed differences in the distribution of the high- and low-EPPI groups (Figure 2H). Kaplan-Meier analysis confirmed a significantly shorter overall survival for the high-EPPI group relative to the low-EPPI group (log-rank *P* < 0.001, Figure 2I). Finally, the AUC values at 1, 3, and 5 years were approximately 0.64, indicating moderate prognostic discrimination in the TCGA-LUAD cohort (Figure 2J).

The EPPI was further evaluated in three independent GEO cohorts. Patients in each cohort were divided into high- and low-EPPI groups using the dataset-specific median EPPI score. Across GSE13213, GSE26939, and GSE29016, the high-EPPI group showed poorer overall survival than the low-EPPI group (Figures 3A–C). Time-dependent ROC analyses further evaluated the prognostic discrimination of EPPI in these independent cohorts (Figures 3A–C). Together, these findings indicate that EPPI reproducibly stratified overall survival across the analyzed LUAD cohorts.

**FIGURE 3:**
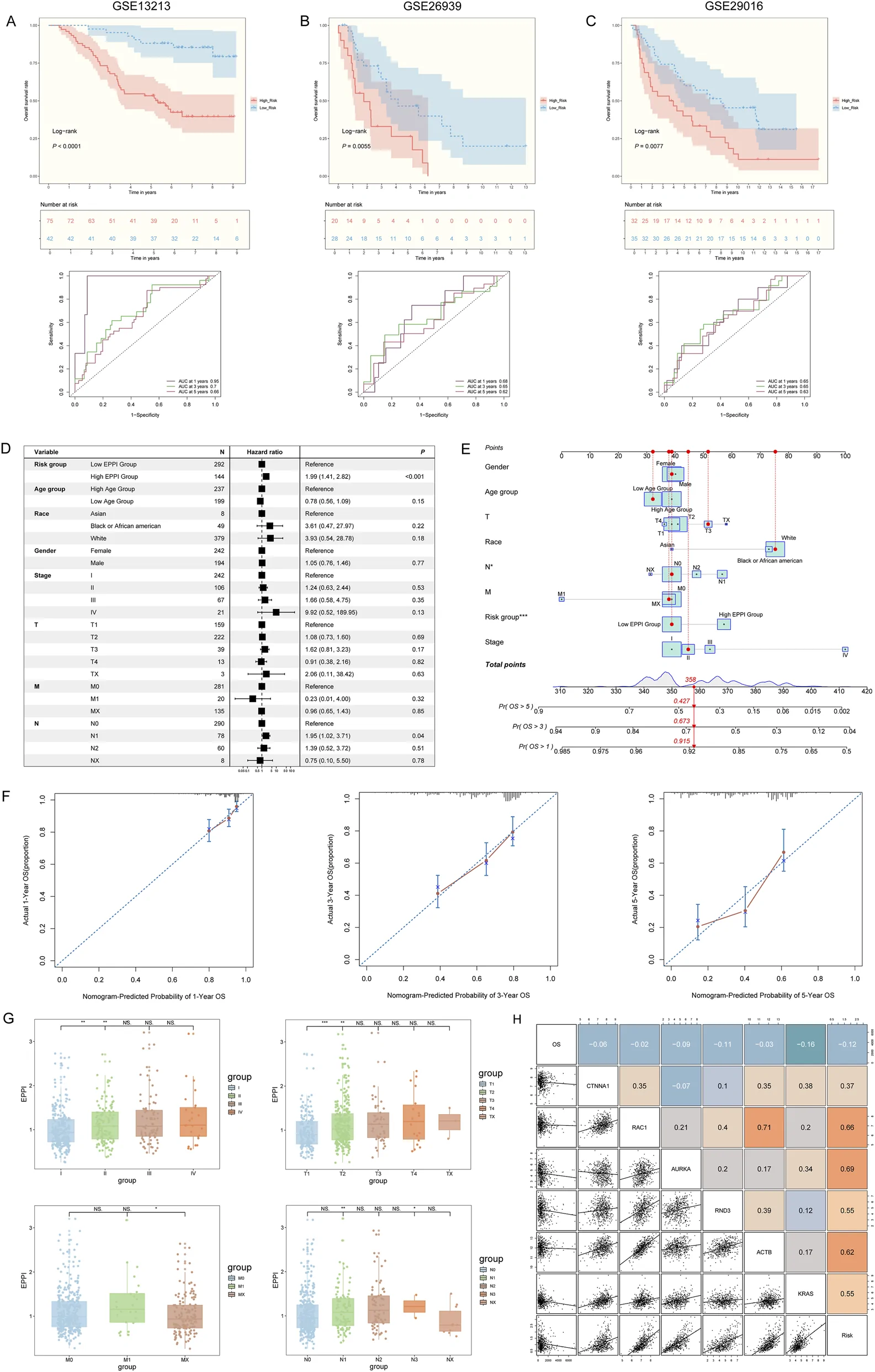
External validation and clinical prognostic assessment of EPPI. **(A–C)** Kaplan–Meier survival curves and time-dependent ROC curves for the external validation cohorts GSE13213, GSE26939, and GSE29016. **(D)** Cox regression analysis of EPPI and clinicopathological variables. **(E)** Nomogram for predicting 1-, 3-, and 5-year overall survival. **(F)** Calibration curves for predicted and observed survival probabilities. **(G)** EPPI scores across clinical stage and T, M, and N categories. **(H)** Correlation matrix of overall survival status, EPPI, and the six model genes. *P < 0.05; **P < 0.01; ***P < 0.001.

### Clinical associations and prognostic value of EPPI

Multivariable Cox regression analysis incorporating EPPI risk group, age, race, sex, overall stage, and T, N, and M categories showed that the high-EPPI group remained significantly associated with poorer overall survival (HR = 1.99, 95% CI: 1.41–2.82, *P* < 0.001; Figure 3D). A nomogram integrating EPPI with these clinicopathological characteristics was constructed to estimate 1-, 3-, and 5-year overall survival probabilities (Figure 3E). The calibration curves showed that the predicted survival probabilities were close to the observed outcomes at 1, 3, and 5 years (Figure 3F). EPPI values also differed according to tumor stage and TNM classification. In general, higher EPPI values were observed in more advanced overall stage, T, and N categories, whereas the pattern across M categories was less consistent (Figure 3G). Correlation analysis showed that EPPI was positively correlated with all six model genes, with the strongest correlations observed for AURKA (r = 0.69), RAC1 (r = 0.66), and ACTB (r = 0.62), followed by RND3 and KRAS (both r = 0.55) and CTNNA1 (r = 0.37) (Figure 3H).

### Functional enrichment analysis between the high- and low-EPPI groups

Differential expression analysis identified substantial gene expression differences between the high- and low-EPPI groups (Figures 4A,B). Gene Ontology evaluation linked the differentially expressed genes primarily to CENP-A-containing nucleosome and chromatin organization, chromosome centromeric core domains, vesicle and granule lumens, antimicrobial humoral responses, and phospholipase-related activities (Figure 4C). Subsequent KEGG enrichment analysis identified pathways encompassing Ras signaling, calcium signaling, neuroactive ligand-receptor interactions, pancreatic secretion, and vascular smooth muscle contraction. This evaluation also highlighted lipid metabolic pathways involving α-linolenic acid, linoleic acid, ether lipid, and arachidonic acid metabolism (Figure 4D). Additional enriched pathways featured systemic lupus erythematosus, neutrophil extracellular trap (NET) formation, transcriptional misregulation in cancer, and viral carcinogenesis.

**FIGURE 4:**
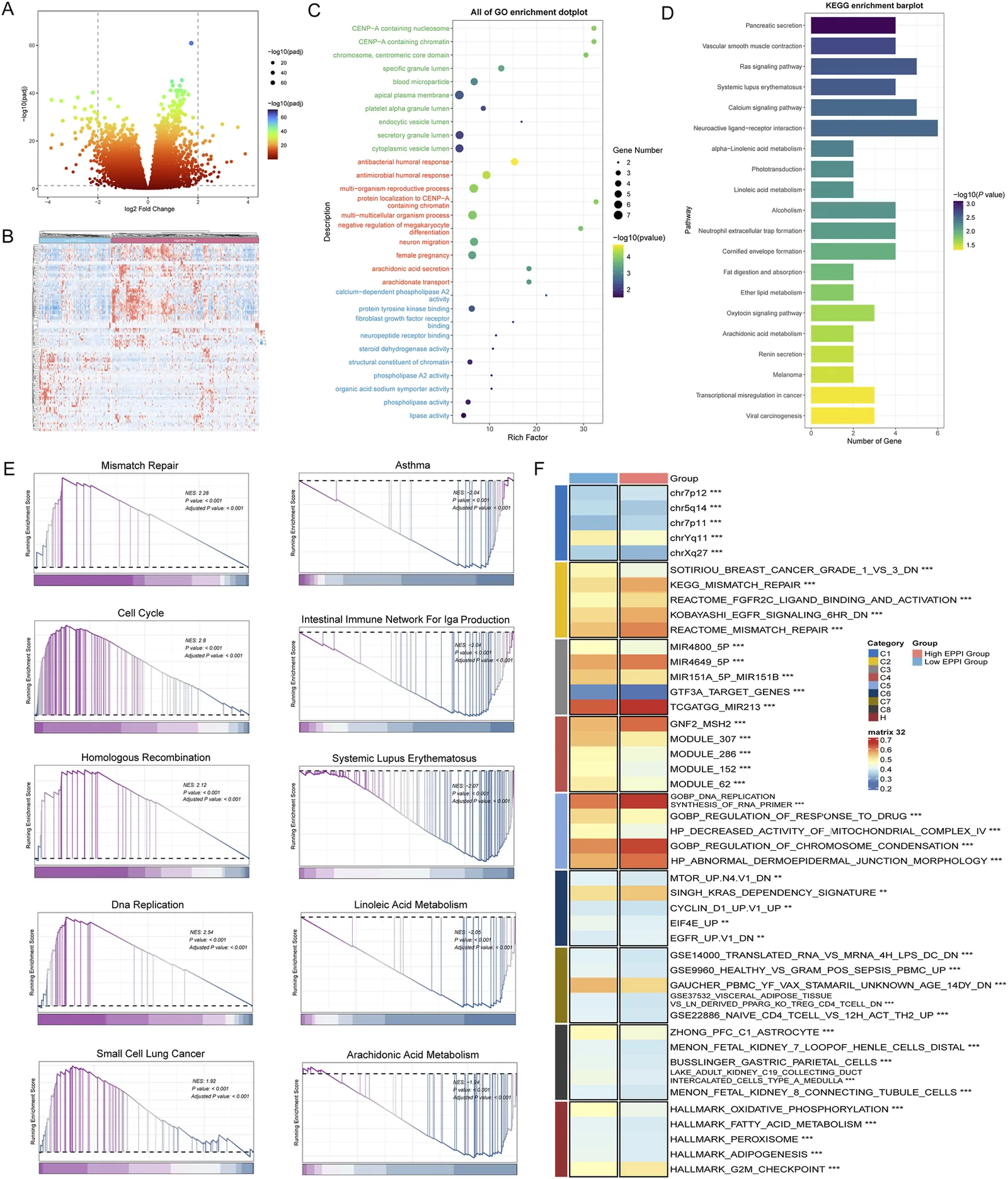
Functional enrichment analysis of the high- and low-EPPI groups. **(A)** Volcano plot of differentially expressed genes. **(B)** Heatmap of differentially expressed genes. **(C)** Gene Ontology enrichment analysis. **(D)** KEGG pathway enrichment analysis. **(E)** Representative GSEA plots comparing the high- and low-EPPI groups. **(F)** Pathway signature analysis of selected oncogenic, functional, and Hallmark gene sets between the high- and low-EPPI groups. C1–C8 represent different signature categories. For oncogenic signatures, “UP” and “DN” follow the original gene-set nomenclature and do not indicate directional regulation in this cohort. *P < 0.05; **P < 0.01; ***P < 0.001.

Gene set enrichment analysis revealed an enrichment of mismatch repair, cell cycle processes, homologous recombination, DNA replication, and small cell lung cancer pathways within the high-EPPI group (Figure 4E). Conversely, asthma, the intestinal immune network for IgA production, systemic lupus erythematosus, linoleic acid metabolism, and arachidonic acid metabolism showed enrichment in the low-EPPI group (Figure 4E). Pathway signature analysis further mapped the differences between the two EPPI groups across selected gene sets related to mismatch repair, DNA replication, FGFR/EGFR and KRAS signaling, mTOR-related signaling, alongside broader metabolic and immune processes (Figure 4F). Collectively, these results indicate that the high-EPPI group features a distinct enrichment of cell-cycle, DNA-replication, and DNA-repair-related gene sets.

### Immune landscape and regulatory molecular profile of the tumor microenvironment

Immune infiltration profiling using ESTIMATE, CIBERSORT, TIMER, EPIC, and xCell showed marked differences in the composition of the tumor microenvironment between the high- and low-EPPI groups (Figure 5A). CIBERSORT further demonstrated substantial intersample heterogeneity in the relative abundance of immune-cell populations (Figure 5B). ESTIMATE analysis showed that the low-EPPI group had higher ESTIMATEScore, ImmuneScore, and StromalScore, whereas the high-EPPI group had higher TumorPurity (Figure 5C). At the level of individual immune-cell populations, increasing EPPI was positively correlated with activated CD4 memory T cells, M0 and M1 macrophages, resting NK cells, and activated mast and dendritic cells, but negatively correlated with plasma cells, resting mast cells, regulatory T cells, and several B-cell populations (Figure 5D). Consequently, the high-EPPI phenotype features a lower overall immune and stromal content paired directly with a distinct redistribution of specific lymphoid and myeloid populations.

**FIGURE 5:**
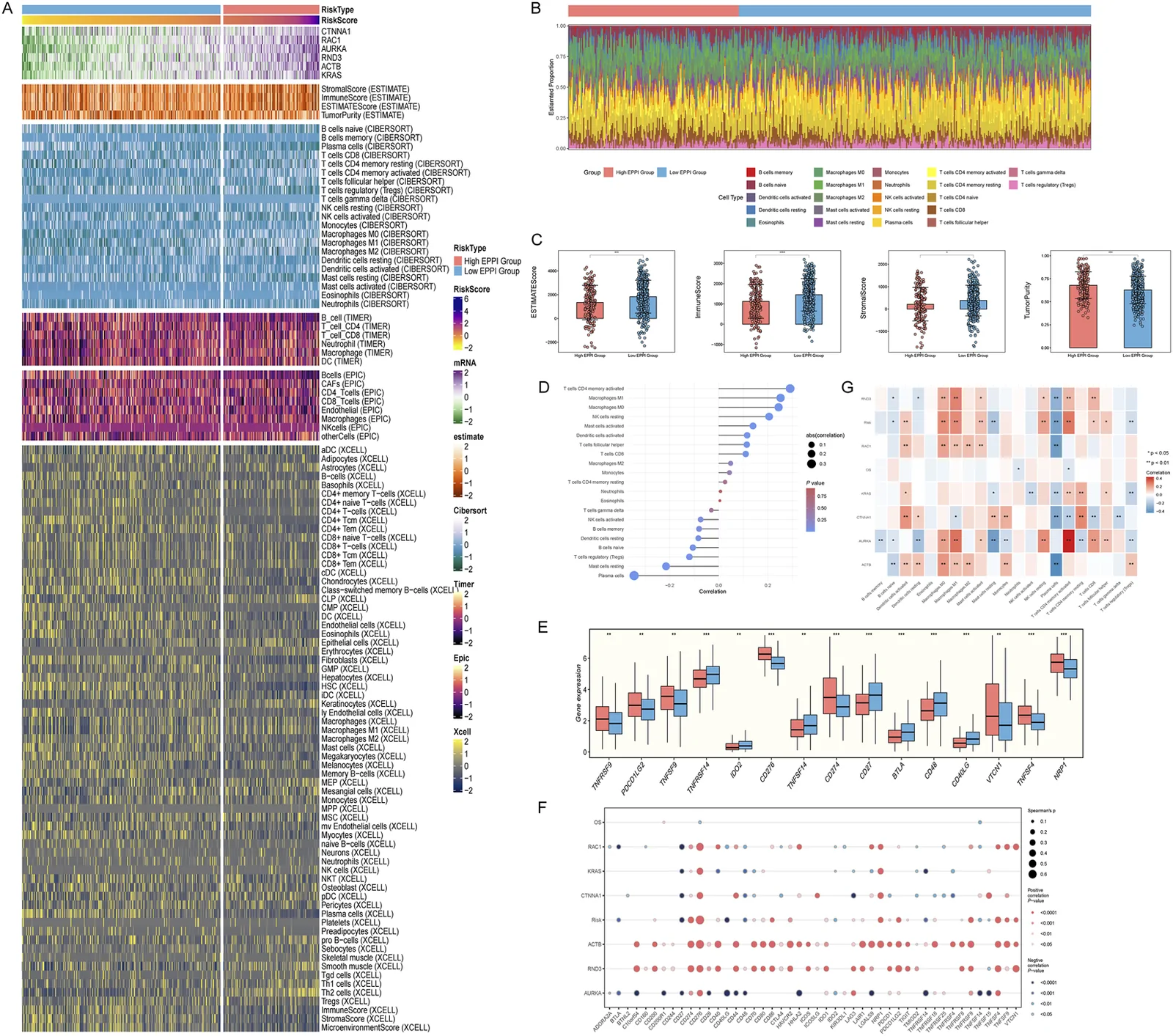
Immune landscape and immune regulatory features of the high- and low-EPPI groups. **(A)** Immune infiltration estimated using ESTIMATE, CIBERSORT, TIMER, EPIC, and xCell. **(B)** Relative proportions of immune-cell populations estimated by CIBERSORT. **(C)** Comparison of ESTIMATEScore, ImmuneScore, StromalScore, and TumorPurity between the high- and low-EPPI groups. **(D)** Correlations between EPPI and immune-cell subsets. **(E)** Expression of immune checkpoint-related genes between the two EPPI groups. **(F)** Correlations among EPPI, the six model genes, and immune regulatory molecules. **(G)** Correlations among EPPI, the six model genes, survival status, and immune infiltration features. *P < 0.05; **P < 0.01; ***P < 0.001.

Immune checkpoint-related genes showed similarly divergent expression patterns between the EPPI groups (Figure 5E). PDCD1LG2, TNFSF9, CD276, CD274, VTCN1, TNFSF4, and NRP1 were expressed at higher levels in the high-EPPI group, whereas TNFRSF14, IDO2, TNFSF14, CD27, BTLA, CD48, and CD40LG were higher in the low-EPPI group (Figure 5E). Correlation analyses further showed different associations of EPPI and the six model genes with immune regulatory molecules and immune-cell infiltration features (Figures 5F,G). Together, these findings characterized the low-EPPI group by greater overall immune and stromal abundance, while higher EPPI was associated with a different distribution of specific immune-cell subsets and immune regulatory molecules.

### Genomic alterations and predicted treatment-related features

Somatic mutation analysis of the TCGA-LUAD cohort showed that 92.05% of tumors harbored somatic mutations, with missense mutations representing the predominant variant type. TP53, TTN, and MUC16 were among the most frequently mutated genes (Figure 6A). Additional characteristics of the mutational landscape, including variant classifications, SNV classes, mutation burden per sample, transition/transversion patterns, and base substitutions, are shown in Supplementary Figure 2. Tumor mutational burden was significantly higher in the high-EPPI group than in the low-EPPI group (Figure 6B). Joint stratification by EPPI and TMB further separated patients into four groups with significantly different overall survival outcomes (Figure 6C). Notably, the high-EPPI group exhibited higher TMB together with poorer survival outcomes.

**FIGURE 6:**
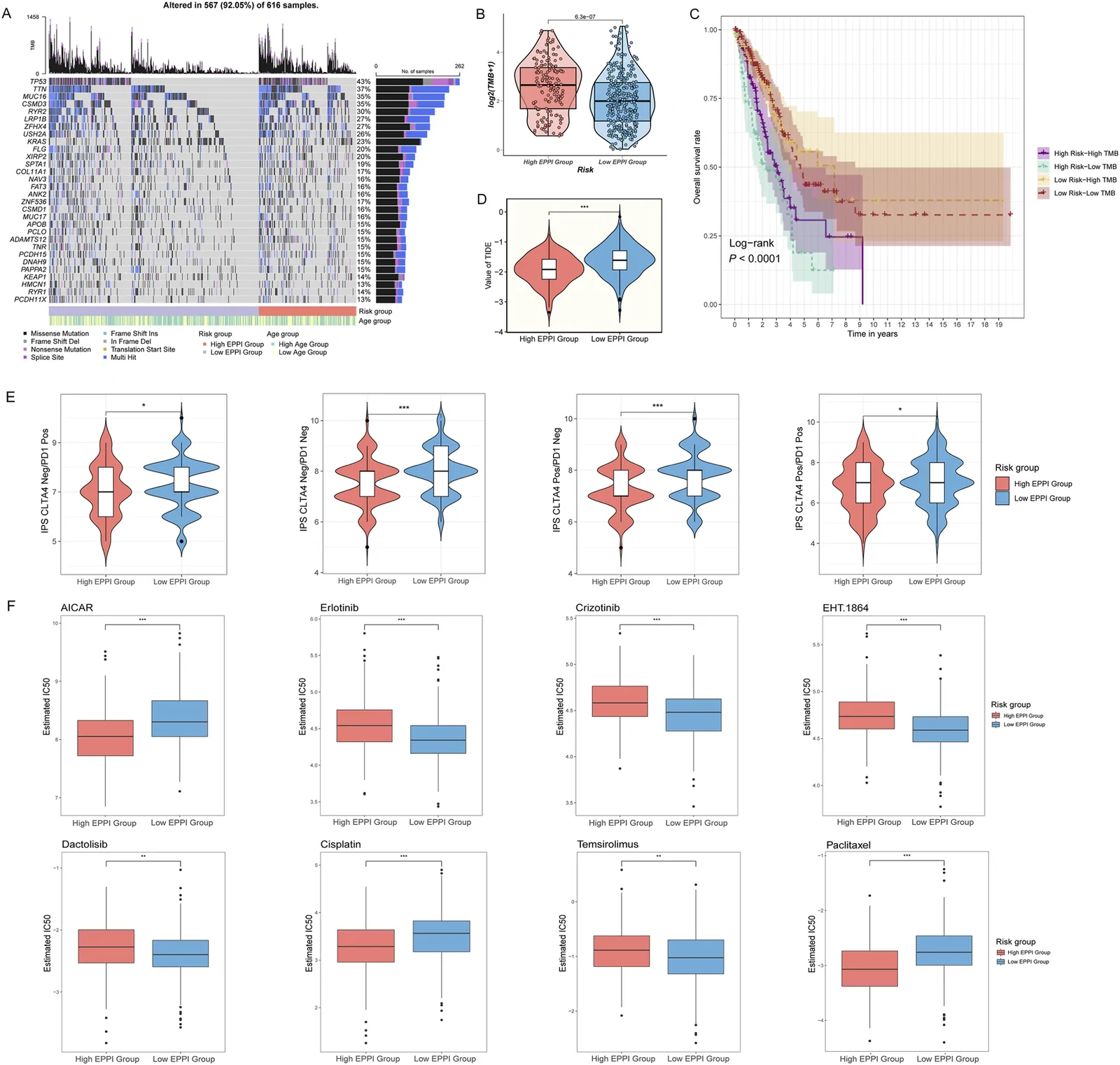
Mutation burden and predicted treatment-related features of the high- and low-EPPI groups. **(A)** Somatic mutation landscape of TCGA-LUAD samples stratified by EPPI. **(B)** Comparison of tumor mutational burden between the high- and low-EPPI groups. **(C)** Kaplan–Meier survival curves based on joint stratification by EPPI and tumor mutational burden. **(D)** Comparison of TIDE scores between the two EPPI groups. **(E)** Immunophenoscores across different CTLA-4/PD-1 settings. **(F)** Predicted IC50 values for selected chemotherapeutic and targeted agents. *P < 0.05; **P < 0.01; ***P < 0.001.

Treatment-related analyses revealed different characteristics between the high- and low-EPPI groups. The high-EPPI group had lower TIDE scores, whereas the low-EPPI group showed higher IPS values across all four evaluated CTLA-4/PD-1 status combinations (Figures 6D,E). These findings showed that the two immunotherapy-related scoring approaches yielded different groupwise profiles across EPPI strata. Drug sensitivity prediction further revealed agent-specific differences between the two groups. Lower predicted IC50 values for AICAR, cisplatin, and paclitaxel were observed in the high-EPPI group, while erlotinib, crizotinib, EHT-1864, dactolisib, and temsirolimus showed lower predicted IC50 values in the low-EPPI group (Figure 6F). Together, these results indicated that EPPI stratification was associated with distinct treatment-related features that varied across immunotherapy-related metrics and individual drugs.

### Molecular docking and spatial transcriptomic characterization of EPPI model genes

Molecular docking was performed for two representative proteins encoded by EPPI model genes. RAC1 was paired with EHT-1864, which was included in the preceding drug-sensitivity analysis, whereas AURKA was evaluated with alisertib as a representative AURKA-targeting compound. RAC1 protein structure 8S1N was docked with EHT-1864, while AURKA protein structure 6C2R was docked with alisertib. The predicted binding energies were −7.2 kcal/mol for RAC1–EHT-1864 and −9.9 kcal/mol for AURKA–alisertib (Supplementary Table S4). Three-dimensional binding conformations and two-dimensional interaction maps illustrated the predicted binding modes of both complexes (Figures 7A,B).

**FIGURE 7:**
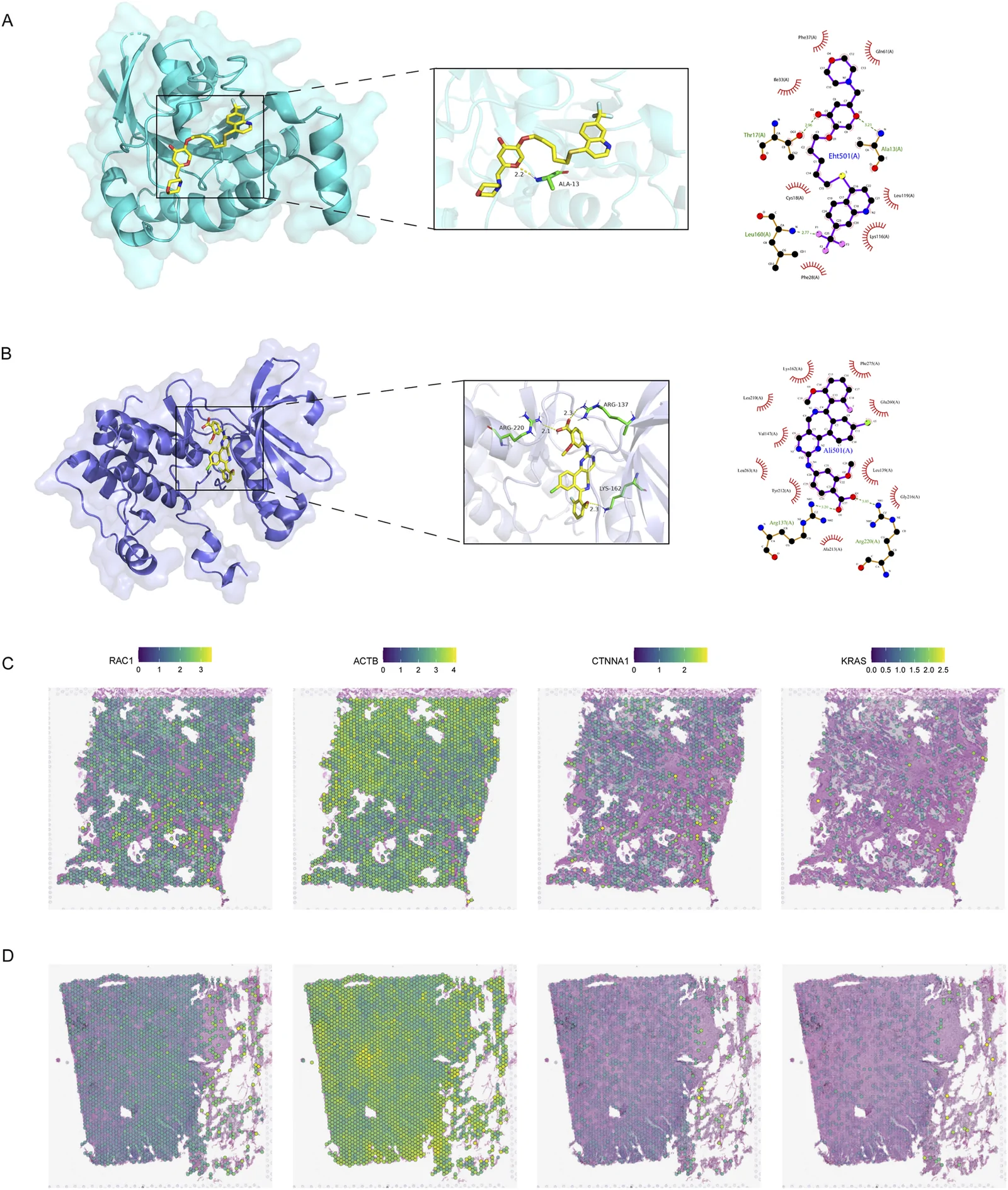
Molecular docking and spatial transcriptomic visualization of representative EPPI model genes. **(A)** Molecular docking analysis of RAC1 with EHT-1864, including the three-dimensional binding conformation, enlarged binding pocket, and two-dimensional interaction diagram. **(B)** Molecular docking analysis of AURKA with alisertib, including the three-dimensional binding conformation, enlarged binding pocket, and two-dimensional interaction diagram. **(C)** Spatial expression patterns of RAC1, ACTB, CTNNA1, and KRAS in LUAD tissue section GSM8427429. **(D)** Spatial expression patterns of RAC1, ACTB, CTNNA1, and KRAS in LUAD tissue section GSM8427438.

Spatial transcriptomic analysis was then used to examine the regional expression patterns of selected EPPI model genes in LUAD tissues. RAC1, ACTB, CTNNA1, and KRAS were detected in both LUAD tissue sections and displayed nonuniform spatial expression across tissue regions (Figures 7C,D).

### Single-cell characterization of EPPI model genes in LUAD

Single-cell transcriptomic analysis identified eight major cell populations in LUAD comprising B cells, T cells, NK cells, mast cells, myeloid cells, epithelial cells, fibroblasts, and endothelial cells (Figures 8A,B). Characteristic lineage-marker expression supported the annotation of these populations, which were distributed across the G1, S, and G2/M cell-cycle phases (Figures 8C,D). Differential expression and functional enrichment analyses linked the immune-cell populations primarily to immune activation and signaling. Conversely, the epithelial, endothelial, and fibroblast clusters exhibited distinct connections to adhesion, migration, morphogenesis, and junction organization (Figures 8E,F).

**FIGURE 8:**
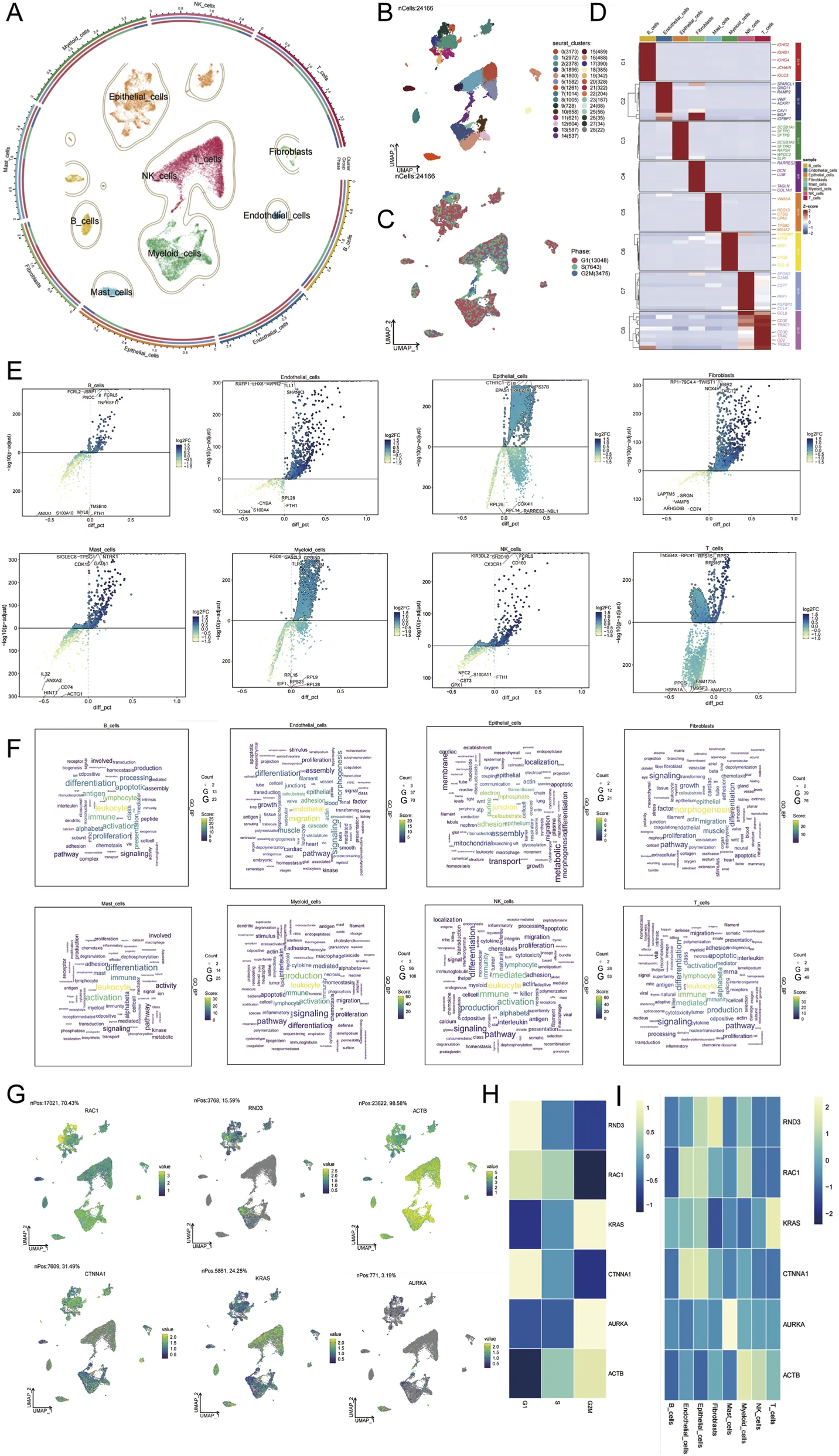
Single-cell landscape and expression architecture of EPPI model genes within LUAD. **(A)** Annotated atlas defining major LUAD lineages. **(B)** UMAP representation of Seurat-derived clusters. **(C)** Cell-cycle phase distribution. **(D)** Heatmap profiling lineage-specific marker genes. **(E)** Volcano representations of differentially expressed genes across lineages. **(F)** Functional term enrichment summaries. **(G)** Feature visualization of the six EPPI model genes. **(H,I)** Heatmap assessments of model gene expression relative to cell cycle progression and cellular lineage identity.

The six EPPI model genes displayed distinct cellular distributions. ACTB was detected in 96.58% of cells and had the broadest distribution. RAC1 followed this wide expression pattern. AURKA was detected in only 3.19% of cells (Figure 8G). RND3 and CTNNA1 expression was higher in G1 cells. KRAS and AURKA expression was higher in G2/M cells (Figure 8H). Among the different cell lineages, CTNNA1 was mainly expressed in epithelial cells. RND3 localized to fibroblasts, KRAS to T cells, and AURKA to mast cells. RAC1 and ACTB maintained a broader distribution across these cell populations (Figure 8I).

### Single-cell characterization of RAC1 in LUAD

RAC1-high and RAC1-low cells were distributed across multiple major LUAD cell populations, showing a broad multi-lineage distribution (Figure 9A). Analysis of stemness, hypoxia, EMT, and tumor progression markers showed higher normalized expression of CTNNB1, EPAS1, HIF1A, and KLF4 in the RAC1-high group, whereas CD44 expression was higher in the RAC1-low group (Figure 9B). RAC1 expression also varied across cell populations, with higher levels in endothelial, epithelial, and myeloid cells and lower levels in B, NK, and T cells (Figure 9C). The two RAC1-defined groups showed distinct RAC1 expression distributions (Figure 9D). RAC1 expression was higher in the G1 and G2/M phases than in the S phase (Figure 9E). Functional annotation linked endothelial, epithelial, and myeloid cells to migration-related processes, oxidative phosphorylation, and immune activation, respectively (Figure 9F). Metabolic scoring showed higher activities of oxidative phosphorylation, glycolysis and gluconeogenesis, glutathione metabolism, riboflavin metabolism, and the pentose phosphate pathway in the RAC1-high group (Figures 9G,H).

**FIGURE 9:**
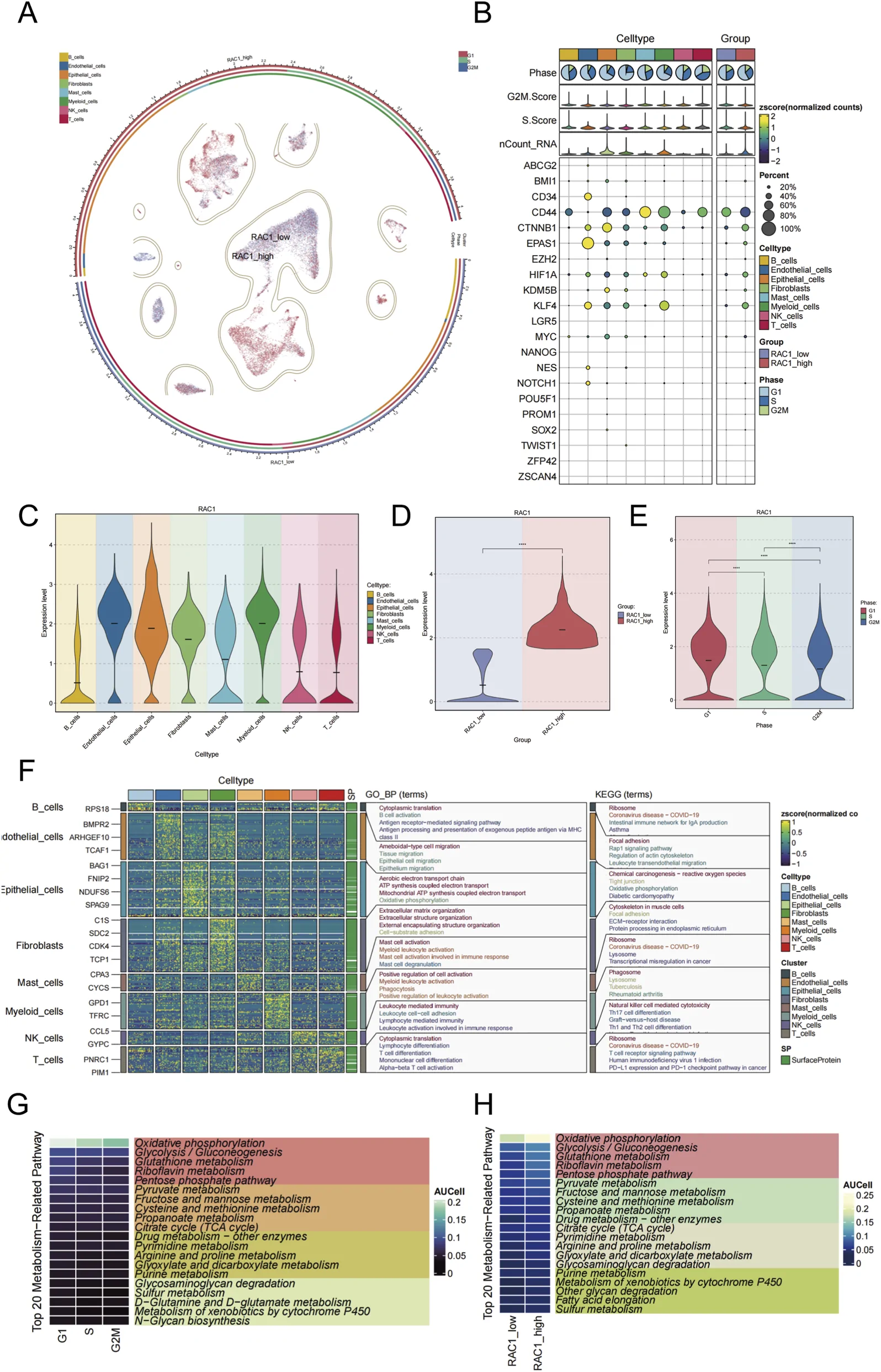
Single-cell expression features of RAC1 in LUAD. **(A)** Single-cell map showing the distribution of RAC1-high and RAC1-low cells. **(B)** Dot plot of stemness-, hypoxia-, EMT-, and tumor progression-related markers. **(C)** RAC1 expression across major LUAD cell populations. **(D)** RAC1 expression between RAC1-high and RAC1-low cells. **(E)** RAC1 expression across G1, S, and G2M phases. **(F)** Heatmap and functional annotation of major LUAD cell populations. **(G,H)** Heatmaps showing metabolism-related pathway scores across cell-cycle phases and RAC1-defined groups. ****P < 0.0001.

### Functional impact of RAC1 depletion on LUAD progression

To assess the functional role of RAC1 in LUAD cells, RAC1 was silenced in A549 and HCC827 cells. qRT-PCR confirmed effective RAC1 knockdown. si-RAC1-1 and si-RAC1-3 showed the greatest silencing efficiency and were selected for subsequent assays (Figure 10A). RAC1 knockdown significantly reduced cell viability, colony formation, and EdU incorporation in both cell lines, indicating decreased proliferative activity (Figures 10B–E). Transwell assays further showed significant reductions in both migration and invasion following RAC1 silencing (Figures 10F,G).

**FIGURE 10:**
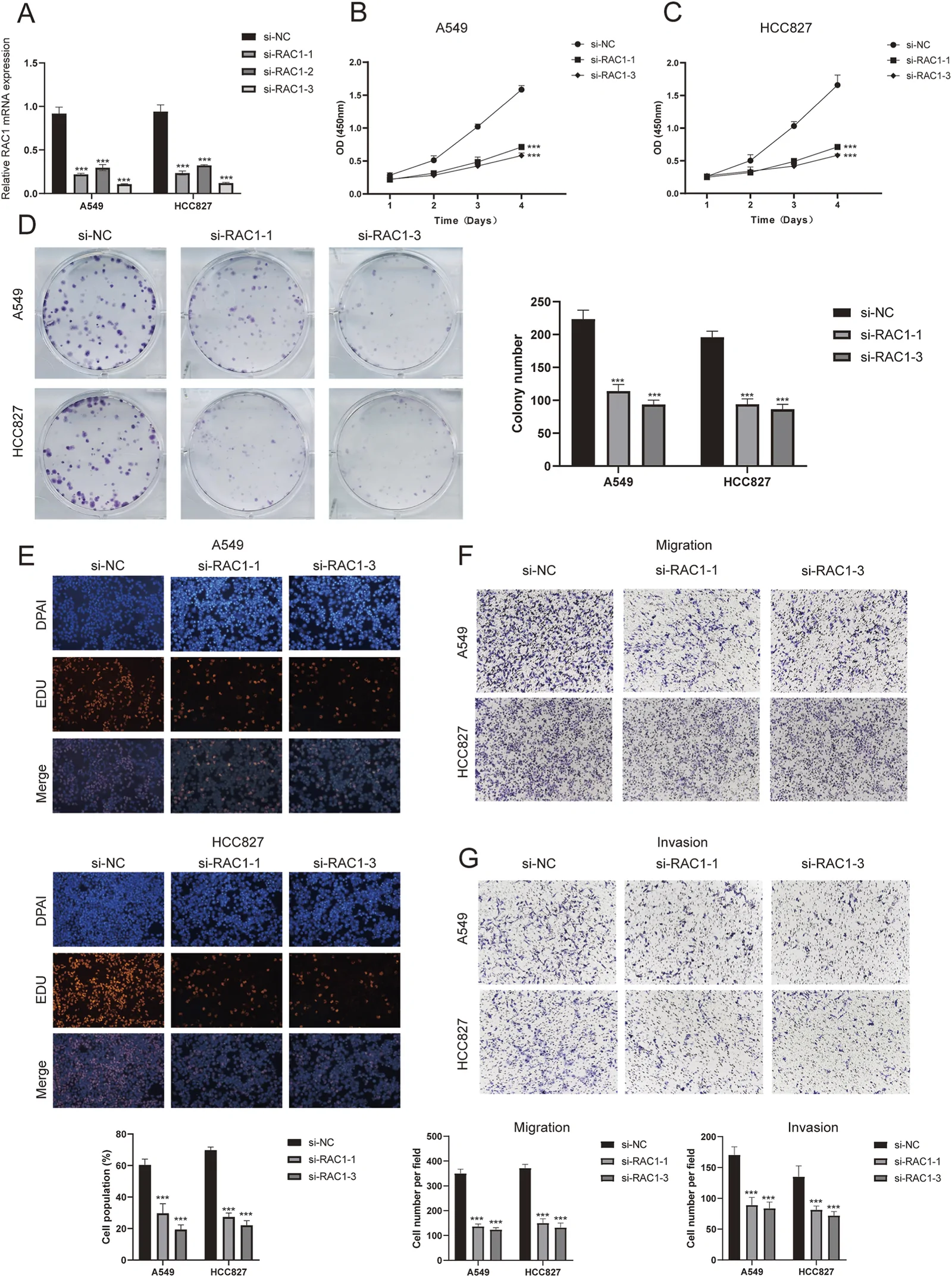
Biological consequences of RAC1 suppression in LUAD. **(A)** qRT-PCR validation of RAC1 knockdown by siRNA. **(B,C)** CCK-8 assays of cell viability. **(D)** Colony formation assay. **(E)** EdU incorporation assay. **(F)** Transwell migration assay. **(G)** Matrigel-coated Transwell invasion assay. Quantitative data are presented as mean ± SD from three independent experiments. ***P < 0.001 compared with the si-NC group.

## Discussion

LUAD is characterized by substantial clinical and molecular heterogeneity, and patients with comparable clinicopathological features may still experience markedly different survival outcomes (Kolb et al., 2024; Faehling et al., 2025). Entosis is a cell-in-cell process in which a viable cell becomes internalized within another cell. Once internalized, the cell may undergo lysosome-mediated degradation, remain viable, divide, or escape. Thus, entotic cell death represents one of several possible outcomes of entosis (Overholtzer et al., 2007; Kim et al., 2024). In this context, the EPPI was constructed from genes involved in cell–cell adhesion, cytoskeletal remodeling, cell-in-cell formation, and the intracellular processing of internalized cells. Across the TCGA-LUAD cohort and independent validation cohorts, EPPI reproducibly stratified overall survival and was also associated with proliferative and DNA-repair-related programs, tumor mutational burden, immune and stromal composition, and predicted treatment-related features.

The association of EPPI with stage and TNM categories indicates that the transcriptional features forming the index are closely related to clinical disease severity. At the same time, EPPI retained a significant association with overall survival after adjustment for clinicopathological variables, suggesting that its prognostic signal provides additional information independent of anatomical stage. This independent association may reflect molecular heterogeneity among tumors with otherwise similar clinical characteristics, highlighting biological differences present within conventional staging groups. When evaluated independently, the moderate time-dependent AUC values indicate that EPPI can serve as a molecular correlate of this prognostic heterogeneity. Incorporating EPPI with clinical variables in the nomogram therefore provides a combined view of molecular and clinicopathological risk information to supplement established staging systems.

Beyond these clinical associations, the functions of the six genes retained in EPPI provide a more specific view of the cellular changes represented by the index. RAC1 participates in actin remodeling and mechanical regulation during cell internalization, whereas KRAS-related signaling has been associated with cell competition during entosis (Sun et al., 2014). In EPPI, KRAS is represented by its mRNA expression, so the KRAS-related signal captured by the index reflects transcript-level variation in KRAS expression. ACTB and CTNNA1 are closely related to the structural requirements of cell-in-cell formation. ACTB is broadly expressed, but as a major component of the actin cytoskeleton, it participates in cell-shape changes and force generation during internalization. CTNNA1 contributes to cadherin–catenin-mediated cell–cell adhesion and the maintenance of intercellular contacts required for this process (Aseervatham, 2020; Lin et al., 2023). RND3 also belongs to the Rho GTPase family, although its functions differ from those of RAC1. In lung cancer, RND3 has been reported to regulate cytoskeletal organization and cell proliferation through the Notch1/NICD/Hes1 pathway (Tang et al., 2014). AURKA is mainly involved in mitotic regulation and cell-cycle progression and is closely associated with aggressive LUAD and poor survival (Galetta and Cortes-Dericks, 2020; Wang et al., 2025). This gene composition links EPPI to cell competition, cell adhesion, and cytoskeletal remodeling involved in cell-in-cell formation, while also incorporating proliferative features associated with aggressive LUAD.

Entosis can have different effects on tumor progression. The degradation of internalized cells may limit tumor-cell survival, whereas cell competition and entosis-associated chromosomal instability may favor tumor progression (Mackay et al., 2018). In the present study, the high-EPPI group showed poorer survival and stronger proliferative activity. Functional enrichment analysis showed that the high-EPPI group exhibited prominent activation of cell-cycle progression, DNA replication, mismatch repair, and homologous recombination. Rapid tumor-cell proliferation increases replicative demand and commonly occurs alongside replication stress, DNA damage, and greater dependence on DNA-repair pathways (Huang and Zhou, 2021). Greater DNA-repair activity may help tumor cells maintain continued growth under replicative pressure, allowing active DNA-repair pathways to occur alongside unfavorable clinical outcomes. AURKA, one of the genes most strongly correlated with EPPI, regulates mitotic spindle assembly and chromosome segregation (Grisetti et al., 2024). Concurrently, E2F-related transcription supports cell-cycle progression and DNA synthesis (Mou et al., 2021; Wang et al., 2022). These findings indicate that the adverse prognostic signal of EPPI is closely associated with a highly proliferative malignant state. The concurrent differences in EMT, KRAS, and EGFR signaling further suggest that high EPPI reflects a broader transcriptional program linked to tumor growth, plasticity, and progression. Thus, the prognostic performance of EPPI is likely to arise in part from established malignant features of LUAD, particularly proliferative activity, operating in addition to entosis-related signals. At the same time, because EPPI originates from genes involved in cell adhesion, cytoskeletal remodeling, Rho GTPase signaling, and cell-in-cell-related processes, these proliferative features may represent one component of a broader entosis-associated tumor state.

The high-EPPI group also showed increased TMB, indicating a greater burden of somatic mutations. As these mutations accumulate, some tumor cells might gain growth or survival advantages that promote tumor progression and contribute to poor survival. Research indicates that entotic engulfment can interfere with host-cell division and promote chromosomal instability (Mackay et al., 2018). Such instability can favor the accumulation of genomic alterations during tumor growth (Zhu et al., 2026).

The low-EPPI group had higher estimated immune and stromal content, whereas tumor purity was higher in the high-EPPI group. Higher tumor purity indicates a larger proportion of tumor cells in the bulk tissue sample and a smaller relative contribution from immune and stromal cells, which can also influence the estimated infiltration levels derived from transcriptomic data. Higher EPPI was also associated with changes in specific immune-cell subsets, checkpoint genes, and NF-κB signaling. Therefore, the lower overall immune and stromal content in the high-EPPI group reflects differences in tumor microenvironment composition, while immune-related signals also vary between the two groups. In LUAD, tumor-cell intrinsic alterations have been reported to regulate immune-related pathways and reshape the tumor microenvironment, thereby influencing disease progression and therapeutic responses (Zhang et al., 2025). TIDE and IPS also showed different patterns. Lower TIDE scores in the high-EPPI group suggested less predicted T-cell dysfunction and exclusion, while the higher IPS in the low-EPPI group indicated greater predicted immunogenicity under the tested checkpoint blockade settings (Jiang et al., 2018; Charoentong et al., 2017). These two scores assess different aspects of antitumor immunity, so their results describe different immune features of the two EPPI groups. Overall, the immune differences between the two EPPI groups involved both cell abundance and immune-related signals.

The poor survival of the high-EPPI group can coexist with its greater predicted sensitivity to cisplatin and paclitaxel. This coexistence may relate to the strong cell-cycle, DNA-replication, and mitotic activity observed in the high-EPPI group, because cisplatin damages DNA and paclitaxel interferes with mitosis in dividing cells. The low-EPPI group showed a different predicted drug-response profile, with lower predicted IC50 values for several targeted agents, including erlotinib, crizotinib, EHT-1864, dactolisib, and temsirolimus. The variation in predicted drug sensitivity between EPPI groups may be related to differences in tumor molecular characteristics, which can influence the response of cancer cells to therapeutic interventions (Guo et al., 2026). The lower predicted IC50 of EHT-1864 in the low-EPPI group was accompanied by a predicted interaction between EHT-1864 and RAC1, one of the EPPI genes. Docking between AURKA and alisertib also showed a predicted interaction, with AURKA closely related to mitotic regulation (Mou et al., 2021; Liu H. et al., 2024). These gene–drug pairs connect the predicted treatment-related differences with specific components of the EPPI and provide candidates for further experimental testing.

EPPI is calculated from RNA obtained from whole tumor tissue. Its value can change with gene expression within cells and with the proportion of different cell types in the sample (Kim et al., 2020; Newman et al., 2019). Single-cell analysis showed that RAC1 and ACTB were expressed across many cell types, whereas other EPPI genes were mainly expressed in selected cell populations. Changes in the proportion of epithelial, immune, stromal, and endothelial cells may therefore change the bulk EPPI score. Spatial transcriptomics also showed that EPPI genes had different expression levels across tumor regions (Wang et al., 2023). A bulk EPPI value combines gene expression from these different regions into one score. Regions with different cell types and different levels of EPPI gene expression may therefore make different contributions to the final score. Entosis requires direct contact between neighboring cells, together with cell adhesion and cytoskeletal remodeling. Differences in local cell composition may affect the direct cell-cell contact required for cell internalization during entosis. The single-cell and spatial analyses therefore show that bulk EPPI values can be influenced by gene expression within cells and by differences in cell composition, while spatial mapping shows that EPPI-gene expression is unevenly distributed across LUAD tissue.

Among the six EPPI genes, RAC1 showed broad expression across bulk, spatial, and single-cell analyses and was associated with cell-cycle and metabolic states. RAC1 also regulates actin remodeling, cell adhesion, and cell movement. In LUAD, RAC1 has been linked to tumor progression (Chen et al., 2023). In A549 and HCC827 cells, RAC1 knockdown reduced proliferation, colony formation, DNA synthesis, migration, and invasion. Despite their different molecular backgrounds, both cell lines showed similar changes after RAC1 knockdown. These results suggest that RAC1 is involved in proliferation, migration, and invasion in both LUAD cell lines. Within the EPPI, RAC1 therefore represents a gene associated with cytoskeletal regulation, cell internalization, and malignant progression.

This study examined the prognostic role and biological features of EPPI derived from entosis-related genes. However, several limitations should be considered. First, the bulk, single-cell, and spatial analyses used retrospective public datasets. Differences in sample size, sequencing platforms, and clinical information may affect the stability of EPPI across patient populations. The current gene set may also cover only part of the molecular processes involved in entosis. Second, bulk EPPI reflects both gene expression within cells and differences in cellular composition, especially for widely expressed genes such as ACTB and RAC1. As a result, changes in tumor-cell expression and changes in tissue composition may be difficult to separate. Third, the links between EPPI and genomic or immune features mainly came from transcriptomic analyses. Direct experimental evidence for the role of entosis in these changes remains limited. The immunotherapy, drug-sensitivity, and molecular docking analyses were also mainly computational. Their treatment value therefore requires further experimental and clinical validation. Finally, RAC1 knockdown affected the tested malignant behaviors in A549 and HCC827 cells. Its specific role in entosis requires further study. Future studies should validate EPPI in larger prospective cohorts and combine clinical data with direct experimental assessment of entosis and treatment response.

## Conclusion

This study developed and validated EPPI as an entosis-informed prognostic index for LUAD. EPPI stratification separated patients with different survival outcomes, tumor proliferative features, immune characteristics, and tissue composition. The findings also show that EPPI captures both entosis-related gene activity and established malignant features of LUAD. These results support further investigation of the clinical relevance of entosis-related molecular changes in LUAD.

## Data Availability

The original contributions presented in the study are included in the article/Supplementary Material, further inquiries can be directed to the corresponding authors.
