# Supplementary material for: An entosis-informed prognostic index for survival stratification and molecular characterization in lung adenocarcinoma
Source: Front Cell Dev Biol. 2026 Oct 5;14:1943127. doi: 10.3389/fcell.2026.1943127 (PMC13677699; doi:10.3389/fcell.2026.1943127)
Supplement: Supplementary file 1 [file Supplementaryfile1.doc]

**Supplementary materials 1**

Supplementary Figure 1. Expression differences of six EPPI model genes between high- and low-EPPI groups. Violin plots showing the expression levels of RAC1, ACTB, AURKA, CTNNA1, RND3, and KRAS in high- and low-EPPI groups.

Supplementary Figure 2. Overview of somatic mutation characteristics in the TCGA-LUAD cohort. **(A)** Summary of mutation classifications, variant types, SNV classes, variants per sample, variant classification distribution, and the top 10 mutated genes. **(B)** Rainfall plot showing the genomic distribution of somatic mutations in a representative LUAD sample. **(C)** Distribution of six base-substitution types, transition/transversion frequency, and mutation composition across samples.
