# Supplementary material for: An entosis-informed prognostic index for survival stratification and molecular characterization in lung adenocarcinoma
Source: Front Cell Dev Biol. 2026 Oct 5;14:1943127. doi: 10.3389/fcell.2026.1943127 (PMC13677699; doi:10.3389/fcell.2026.1943127)
Supplement: Supplementary file 2 [file Supplementaryfile2.doc]

**Supplementary materials 2**

# **Table S1. Collection and functional annotation of entosis-related genes used for EPPI construction**

| **Gene symbol** | **Entosis-related category** | **Biological relevance** |
| --- | --- | --- |
| ACTB | Actomyosin remodeling | Core actin cytoskeletal component involved in mechanical regulation |
| AR | Cell signaling regulation | Associated with tumor-related signaling pathways |
| ARHGAP35 | Rho GTPase signaling | Regulates Rho family GTPase activity |
| ATG5 | Autophagy and intracellular degradation | Participates in autophagy-related degradation processes |
| ATG7 | Autophagy and intracellular degradation | Required for autophagosome formation |
| AURKA | Cell-cycle regulation | Regulates mitotic progression |
| BECN1 | Autophagy and lysosomal regulation | Regulates autophagy initiation |
| CDC42 | Rho GTPase signaling/cytoskeletal remodeling | Regulates actin dynamics and cell morphology |
| CDH1 | Cell adhesion | Mediates epithelial cell-cell adhesion |
| CDH3 | Cell adhesion | Participates in cadherin-mediated interactions |
| CTNNA1 | Cell adhesion/junction organization | Links cadherin complexes with actin cytoskeleton |
| CTNNB1 | Cell adhesion/signaling | Component of cadherin-catenin complexes |
| CTNND1 | Cell adhesion | Regulates cadherin-mediated junctions |
| CTTN | Actin remodeling | Regulates cortical actin organization |
| CXCL8 | Inflammatory signaling | Associated with tumor microenvironment regulation |
| CYBB | Cellular signaling/oxidative regulation | Associated with cellular stress responses |
| DIAPH1 | Actomyosin remodeling | Regulates actin polymerization |
| EIF4EBP1 | Translational regulation | Controls protein synthesis responses |
| EIF4EBP2 | Translational regulation | Regulates translation initiation |
| EZR | Cytoskeletal organization | Links membrane proteins with actin cytoskeleton |
| FOXO1 | Stress response regulation | Regulates survival and metabolic responses |
| GZMB | Cell death regulation | Associated with immune-mediated cell elimination |
| JUP | Cell adhesion | Component of cell junction complexes |
| KIF2C | Cell-cycle regulation | Involved in mitotic regulation |
| KRAS | Growth signaling regulation | Regulates oncogenic signaling pathways |
| LAMP1 | Lysosomal processing | Regulates lysosomal function |
| LPAR2 | Cell signaling regulation | Participates in survival signaling |
| MAP1LC3A | Autophagy regulation | Involved in autophagosome formation |
| MCOLN1 | Lysosomal function | Regulates lysosomal ion homeostasis |
| MRTFA | Cytoskeletal transcription regulation | Links actin dynamics with transcription |
| MTOR | Growth signaling/autophagy regulation | Integrates growth and metabolic signals |
| MTUS2 | Cellular signaling regulation | Related to intracellular signaling |
| MYH14 | Actomyosin remodeling | Involved in myosin-dependent regulation |
| MYH9 | Actomyosin remodeling | Regulates mechanical processes |
| MYL2 | Actomyosin remodeling | Involved in contractility |
| ORAI1 | Calcium signaling | Involved in calcium-dependent responses |
| PCK2 | Metabolic regulation | Associated with metabolic adaptation |
| PIK3C3 | Vesicle/autophagy regulation | Participates in intracellular trafficking |
| PIKFYVE | Vesicle regulation | Participates in endosomal trafficking |
| PRKAA1 | AMPK signaling | Regulates energy and stress responses |
| PRKAB1 | AMPK signaling | Regulates energy and stress responses |
| PRKAG1 | AMPK signaling | Regulates energy and stress responses |
| PTK2 | Cell adhesion/mechanotransduction | Regulates adhesion signaling |
| RAC1 | Rho GTPase signaling/cytoskeletal remodeling | Regulates actin dynamics and cell interaction |
| RHOA | Rho GTPase signaling | Regulates actomyosin contraction |
| RNF146 | Cellular signaling regulation | Involved in protein regulation |
| ROCK1 | Rho pathway/cytoskeletal remodeling | Regulates actomyosin contractility |
| ROCK2 | Rho pathway/cytoskeletal remodeling | Regulates actomyosin contractility |
| RND3 | Rho family regulation/cytoskeletal remodeling | Modulates Rho signaling |
| RUBCN | Autophagy regulation | Modulates autophagic pathways |
| SCARNA5 | RNA regulation | Related to cellular regulation |
| SRF | Cytoskeletal transcription regulation | Controls actin-related gene expression |
| TNFSF10 | Cell death regulation | Participates in regulated cell death signaling |
| TP53 | Stress response regulation | Regulates cellular stress responses |
| UVRAG | Autophagy/lysosomal regulation | Involved in autophagosome maturation |
| VCL | Cell adhesion/cytoskeletal linkage | Links adhesion structures with actin cytoskeleton |

**Table S2. Primer sequences used for qRT-PCR**

| **Gene** | **Forward Primer (5′–3′)** | **Reverse Primer (5′–3′)** |
| --- | --- | --- |
| RAC1 | CGGTGAATCTGGGCTTATGGGA | AGGATGTTGCTGTCGTCAAAC |
| GAPDH | GTCTCCTCTGACTTCAACAGCG | GGAGGTTATATCCTTACCGTACG |

**Table S3. Features and coefficients of the entosis-related prognostic model developed for LUAD.**

| **Gene** | **Coefficient** | **HR** | **HR.95% CI** | ***P*-value** |
| --- | --- | --- | --- | --- |
| CTNNA1 | 0.111 | 1.1 | (0.86-1.5) | 0.41 |
| RAC1 | 0.205 | 1.2 | (0.83-1.8) | 0.3 |
| AURKA | 0.184 | 1.2 | (1-1.4) | 0.012 |
| RND3 | 0.078 | 1.1 | (0.94-1.2) | 0.27 |
| ACTB | 0.161 | 1.2 | (0.81-1.7) | 0.4 |
| KRAS | 0.107 | 1.1 | (0.9-1.4) | 0.33 |

**Table S4. Molecular docking results of selected EPPI targets**

| **Target** | **PDB ID** | **Compound** | **PubChem CID** | **Binding energy** |
| --- | --- | --- | --- | --- |
| RAC1 | 8S1N | EHT-1864 | 9938202 | -7.2 kcal/mol |
| AURKA | 6C2R | Alisertib | 24771867 | -9.9 kcal/mol |
